# The structures of cytosolic and plastid-located glutamine synthetases from *Medicago truncatula* reveal a common and dynamic architecture

**DOI:** 10.1107/S1399004713034718

**Published:** 2014-03-19

**Authors:** Eva Torreira, Ana Rita Seabra, Hazel Marriott, Min Zhou, Óscar Llorca, Carol V. Robinson, Helena G. Carvalho, Carlos Fernández-Tornero, Pedro José Barbosa Pereira

**Affiliations:** a Chemical and Physical Biology, Centro de Investigaciones Biológicas – CSIC, Ramiro de Maeztu 9, 28040 Madrid, Spain; b Molecular Biology of Nitrogen Assimilation, IBMC – Instituto de Biologia Molecular e Celular, Universidade do Porto, Rua do Campo Alegre 823, 4150-180 Porto, Portugal; c Chemistry Research Laboratory, University of Oxford, South Parks Road, Oxford OX1 3QZ, England; d Biomolecular Structure Group, IBMC – Instituto de Biologia Molecular e Celular, Universidade do Porto, Rua do Campo Alegre 823, 4150-180 Porto, Portugal

**Keywords:** glutamate-ammonia ligase, leguminous, herbicide resistance, *Medicago truncatula*

## Abstract

The first step of nitrogen assimilation in higher plants, the energy-driven incorporation of ammonia into glutamate, is catalyzed by glutamine synthetase. This central process yields the readily metabolizable glutamine, which in turn is at the basis of all subsequent biosynthesis of nitrogenous compounds. The essential role performed by glutamine synthetase makes it a prime target for herbicidal compounds, but also a suitable intervention point for the improvement of crop yields. Although the majority of crop plants are dicotyledonous, little is known about the structural organization of glutamine synthetase in these organisms and about the functional differences between the different isoforms. Here, the structural characterization of two glutamine synthetase isoforms from the model legume *Medicago truncatula* is reported: the crystallographic structure of cytoplasmic GSII-1a and an electron cryomicroscopy reconstruction of plastid-located GSII-2a. Together, these structural models unveil a decameric organization of dicotyledonous glutamine synthetase, with two pentameric rings weakly connected by inter-ring loops. Moreover, rearrangement of these dynamic loops changes the relative orientation of the rings, suggesting a zipper-like mechanism for their assembly into a decameric enzyme. Finally, the atomic structure of *M. truncatula* GSII-1a provides important insights into the structural determinants of herbicide resistance in this family of enzymes, opening new avenues for the development of herbicide-resistant plants.

## Abstract 2

The experimental models of dicotyledonous cytoplasmic and plastid-located glutamine synthetases unveil a conserved eukaryotic-type decameric architecture, with subtle structural differences in *M. truncatula* isoenzymes that account for their distinct herbicide resistance.

## 1. Introduction   

Glutamine synthetase (GS; EC 6.3.1.2) is a vital enzyme in the nitrogen metabolism of all living systems. GS catalyzes the first step in nitrogen assimilation, the ATP-dependent ligation of ammonia and glutamate to yield glutamine (1), which is then used for the biosynthesis of essentially all nitrogenous compounds in the cell (Lea & Miflin, 2010 ▶),
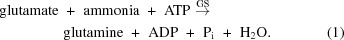



The assimilation of ammonia is important for all organisms, but it is of special interest in plants, both because it is the main entry route of inorganic nitrogen into organic composition and because nitrogen is a major limiting factor in plant growth and productivity. The reaction catalyzed by GS is a key component of plant nitrogen-use efficiency (NUE) and plant yield, and an increase in GS activity can potentially enhance plant productivity (Lea & Miflin, 2010 ▶). Conversely, GS inhibition leads to plant death, and a number of herbicides have been designed on this principle. An in-depth understanding of the molecular details of glutamine synthetase activity is therefore of crucial importance not only to improve NUE but also for the design of herbicide-resistant plants.

Glutamine synthetase is a fascinating, complex and highly regulated enzyme found in all living organisms (Kumada *et al.*, 1993 ▶), but GS proteins differ significantly in molecular mass, sequence and quaternary structure among the different forms of life. Three major classes, referred to as GSI, GSII and GSIII, have been described (Woods & Reid, 1993 ▶). GSI, the best-characterized class, includes enzymes typically found in prokaryotes that display a homododecameric arrangement of subunits with masses ranging from 44 to 60 kDa. Following the groundbreaking determination of the atomic structure of GlnA from *Salmonella typhimurium* by X-ray crystallography (Almassy *et al.*, 1986 ▶), the structures of GSI from *Mycobacterium tuberculosis* (Gill *et al.*, 2002 ▶) and *Synechocystis* sp. (PDB entry 3ng0; L. Saelices, D. Cascio, F. J. Florencio & M. I. Muro-Pastor, unpublished work) have greatly contributed to current knowledge of the structural features and enzymology of GS. Typical eukaryotic GSs belong to class II, forming homodecamers of 35–50 kDa subunits. The first three-dimensional GSII structure determined was that from maize (Unno *et al.*, 2006 ▶), followed by the mammalian (Krajewski *et al.*, 2008 ▶) and yeast (He *et al.*, 2009 ▶) enzymes, all of which are present in the cytoplasm. Finally, GSIII has been identified in cyanobacteria (Reyes & Florencio, 1994 ▶) and two unrelated anaerobic bacteria (Southern *et al.*, 1986 ▶; Goodman & Woods, 1993 ▶). Although its 75–83 kDa subunits were thought to be organized as homohexamers (Reyes & Florencio, 1994 ▶), the crystal structure of *Bacteroides fragilis* GSIII revealed a dodecameric architecture showing significant structural differences from GSI (van Rooyen *et al.*, 2011 ▶). Interestingly, genes encoding prokaryotic-type GSI-like molecules have also been found in plants (Mathis *et al.*, 2000 ▶; Doskočilová *et al.*, 2011 ▶) and mammals, and display a structural organization similar to their bacterial homologues, as confirmed by the electron-microscopy structure of mouse lengsin (Wyatt *et al.*, 2006 ▶). On the other hand, a few soil-dwelling bacteria (belonging to the Rhizobiaceae, Frankiaceae and Strepto­mycetaceae families) contain an eukaryotic-type GS (Carlson & Chelm, 1986 ▶). Whereas the function of these proteins is still unclear, they probably result from lateral gene transfer during evolution (Ghoshroy *et al.*, 2010 ▶).

The overall geometry of the active site is the most conserved structural element amongst GS enzymes (Eisenberg *et al.*, 2000 ▶; Unno *et al.*, 2006 ▶; van Rooyen *et al.*, 2011 ▶). This funnel-shaped pocket is formed by the N-terminal β-­grasp domain of one monomer and the highly curved β-­sheet in the C-terminal catalytic domain of the neighbouring monomer. The pocket closes upon nucleotide binding and several additional changes occur upon glutamate attachment (Krajewski *et al.*, 2008 ▶). Despite conservation of the active-site architecture, eukaryotic GSII does not contain the adenylation loop which regulates GS activity in bacteria. Another remarkable structural difference between GSI and GSII is the inter-ring association. While hexameric GSI rings tightly interact by insertion of the C-terminal helix of each monomer into a hydrophobic cavity on the corresponding subunit of the opposite hexameric ring, GSII rings interact weakly and only through internal loops.

In plants, the ammonia for GS activity may be derived from primary sources (nitrogen fixation, nitrate assimilation and ammonia uptake) or from secondary metabolism such as photorespiration and amino-acid catabolism; therefore, the enzyme plays essential roles in both primary nitrogen assimilation and nitrogen recycling (Bernard & Habash, 2009 ▶; Lea & Miflin, 2010 ▶). Consistent with the diversity of metabolic roles, GS exists in plants as a number of isoenzymes, located both in the cytosol (GSII-1) and in the plastids (GSII-2), that are encoded by a small gene family. Each of the GS genes displays distinct patterns of expression in different organs of the plant and their encoded enzymes participate in different metabolic processes. In the model legume *Medicago truncatula*, the GS gene family consists of four expressed genes, *MtGSII-1a*, *MtGSII-1b*, *MtGSII-2a* and *MtGSII-2b* (Stanford *et al.*, 1993 ▶; Carvalho, Lescure *et al.*, 2000 ▶; Carvalho, Lima *et al.*, 2000 ▶; Melo *et al.*, 2003 ▶; Seabra *et al.*, 2010 ▶), the latter of which is unique to *M. truncatula* and closely related species. The other three GS genes are expressed in almost all organs of the plant, but in a cell-specific manner. *MtGSII-1a* is expressed in the vascular bundles of all plant organs (Carvalho, Lescure *et al.*, 2000 ▶; Carvalho, Lima *et al.*, 2000 ▶) and is highly up­regulated in the nodules, accounting for the production of over 90% of the total nodule GS activity. The *MtGSII-1a*-encoded isoenzyme is responsible for the assimilation of ammonia released by nitrogen fixation (Carvalho, Lima *et al.*, 2000 ▶). The enzyme encoded by *MtGSII-1b* appears to play a housekeeping role, being expressed at lower levels and almost everywhere in the plant, whereas *MtGSII-2a* is highly expressed in all photosynthetic tissues and is mainly responsible for the reassimilation of ammonia released by photorespiration (Carvalho & Cullimore, 2003 ▶). Finally, MtGSII-2b is exclusively expressed during seed filling and its physiological role is probably therefore related to protein storage (Seabra *et al.*, 2010 ▶).

The specialization of closely related isoenzymes to a particular metabolic context certainly reflects specific functional determinants that make them adapt better to each environmental condition. However, very little is known regarding the structural features and catalytic determinants of the different isoenzymes. The only plant GS enzyme structurally characterized to date is maize cytosolic ZmGSII-1a, which proved to be a decamer (Unno *et al.*, 2006 ▶). However, an octameric architecture has been suggested for cytosolic GSII from *Phaseolus vulgaris* (Llorca *et al.*, 2006 ▶; Betti *et al.*, 2012 ▶), while the structure of plastid-located GS is unknown. Comparison of experimental models of closely related proteins can assist in the identification of critical structural components responsible for enhancement of activity and in the design of herbicide-resistant enzymes.

Here, we present the unique crystallographic structure of a cytosolic GS from a dicotyledonous plant, the model legume *M. truncatula*. We also present the electron cryomicroscopy (cryo-EM) structure of its plastid-located GS enzyme and demonstrate that it similarly organizes in homodecamers composed of two pentameric rings. We show that these rings associate weakly and propose a dynamic zipper-like mechanism for inter-ring assembly. A comparison between these structures leads us to identify common features in enzymes that function under different biochemical contexts and differ in catalytically properties, and provides insights into the structural determinants of herbicide resistance.

## 2. Methods   

### 2.1. Recombinant protein expression and purification   

The cloning procedures for N-terminally His-tagged proteins have been described previously (Seabra *et al.*, 2009 ▶). The cDNAs coding for *M. truncatula* MtGSII-1a and MtGSII-1b and the sequences coding for the mature MtGSII-2a and MtGSII-2b polypeptides were amplified by PCR from the vector pTrc99A (Carvalho *et al.*, 1997 ▶; Melo *et al.*, 2003 ▶; Seabra *et al.*, 2010 ▶) using the forward primer 5′-TTGACAATTAAT­CATCCGGC-3′ and the specific reverse primers 5′-TGG­TTGTGGTCGACTGGTTTCC-3′ (MtGSII-1a), 5′-AGCGTGGTGTCGACTGGTTTCC-3′ (MtGSII-1b), 5′-AATAGA­TGTCGACTTTCAATGC-3′ (MtGSII-2a) and 5′-AATAGATGTCGACCTTCAATGC-3′ (MtGSII-2b). The same forward primer, which annealed in the vector backbone, was used to amplify the four cDNAs. The reverse primers were designed to specifically abolish the stop codon of each GS cDNA and to include the *Sal*I restriction sequence. The PCR fragments were partially digested with the restriction enzyme *Nco*I and fully digested with *Sal*I. The PCR digests were cloned into the *Nco*I and *Xho*I sites of pET-24-d-T vector, a derivative of pET-24-d(+) (Novagen) containing a C-terminal His tag and a thrombin cleavage site. The resulting plasmids encode C-­terminally His_6_-tagged GS fusion proteins in which the sequence LVPRGSVEHHHHHH follows the GS coding sequences. The four expression constructs were sequenced.

To express the His-tagged GS proteins, *Escherichia coli* BL21 CodonPlus (DE3) RIL cells (Stratagene) harbouring the pET-24d-GS plasmids were first cultured in LB medium at 37°C to mid-exponential growth (OD_600_ = 0.5). Expression of the recombinant proteins was then induced with IPTG (final concentration 1 m*M*) and cell growth continued overnight at 20°C. The cells were harvested by centrifugation at 2800*g*, resuspended in extraction buffer (10 m*M* HEPES buffer pH 7.4, 10 m*M* magnesium sulfate, 5 m*M* glutamate, 500 m*M* NaCl, 20 m*M* imidazole), disrupted by sonication and centrifuged (60 min, 38 000*g*, 4°C) to remove cell debris. The crude protein extract was filtered through a 5 µm low-protein-binding filter and loaded onto a 5 ml Ni-Sepharose column (GE Healthcare) equilibrated with buffer *A* (10 m*M* HEPES buffer pH 7.4, 500 m*M* NaCl, 20 m*M* imidazole). Elution of the bound fusion protein was achieved by increasing the imidazole concentration in buffer *A* to 230 m*M*. The GS-containing fractions were pooled, dialyzed against 10 m*M* HEPES PH 7.4 and concentrated to 5 mg ml^−1^ on a centrifugal concentration device with a 10 kDa molecular-weight cutoff membrane (Vivaproducts).

### 2.2. Crystallographic structure determination and refinement   

*M. truncatula* GSII-1a was crystallized in space group *P*2_1_ and a complete 2.35 Å resolution X-ray diffraction data set was collected from a single cryocooled crystal on ESRF beamline ID14-EH2 as described previously (Seabra *et al.*, 2009 ▶). The data were processed with *MOSFLM* (Leslie & Powell, 2007 ▶) and scaled with *SCALA* (Evans, 2006 ▶) from the *CCP*4 suite (Winn *et al.*, 2011 ▶). The structure was solved by molecular replacement with *Phaser* (McCoy *et al.*, 2007 ▶) using the coordinates of *Zea mays* GS1a (Unno *et al.*, 2006 ▶) as search model with all non-identical nonglycine residues truncated to alanine (Seabra *et al.*, 2009 ▶).

The model was iteratively improved by alternating cycles of manual model building with *Coot* (Emsley *et al.*, 2010 ▶) and refinement with *CNS* (Brunger, 2007 ▶). The resulting isotropic model was further refined using TLS parameters and torsion-based NCS restraints as implemented in *PHENIX* (Afonine *et al.*, 2012 ▶). The refinement statistics are summarized in Table 1 ▶. The final model has good stereochemistry, with 100% of the residues in the allowed regions of the Ramachandran plot. It comprises residues Met1–Tyr284 and Asn302–Pro356 in chain *A*, Met1–His277, Val315–Val317 and Tyr328–Leu353 in chain *B*, Met1–Lys278, Ser314–Val317 and Tyr328–Pro356 in chain *C*, Met1–Arg276, Ser314–Val317, Gly327–Asp331 and Met338–Pro356 in chain *D*, Met1–Glu259, Ile265–Arg276, Phe329–Asp331 and Met338–Pro356 in chain *E*, Met1–His277, Ser314–Val317, Gly327–Arg332 and Asn337–Pro356 in chain *F*, Met1–His277, Ser314–Val317 and Gly327–Pro356 in chain *G*, Met1–His277, Tyr328–Arg332 and Asn337–Pro356 in chain *H*, Met1–Arg276, Ser314–Val317, Tyr328–Asp331 and Met338–Pro356 in chain *I* and Met1–Arg276, Gly327–Arg332 and Asn337–Pro356 in chain *J* of the glutamine synthetase decamer present in the asymmetric unit. There was no interpretable electron density for the remaining amino-acid residues, including the totality of the N-terminal affinity tags, which were therefore excluded from the model. The refined coordinates and structure factors were deposited in the PDB (http://www.pdb.org) under accession code 4is4.

### 2.3. Electron microscopy   

Purified GSII-2a (0.3 mg ml^−1^) was vitrified using glow-discharged QUANTIFOIL R 2/2 holey grids and a Vitrobot (Gatan) according to previously described methods (Dubochet *et al.*, 1988 ▶). Frozen grids were transferred to a JEM-2200FS microscope at low temperature (−170°C) using a cryo-transfer holder (Gatan). The microscope was operated under cryogenic conditions at an accelerating voltage of 200 kV. Data were collected under low-dose conditions on a 4k × 4k CCD (Gatan) at a magnification of 83 393, with a pixel size of 1.62 Å at the specimen level. The contrast transfer function of the micrographs was determined with *ctffind*3 (Mindell & Grigorieff, 2003 ▶). 7111 particle images were selected manually with *boxer* from the *EMAN* package (Ludtke *et al.*, 1999 ▶). Reference-free two-dimensional alignment of the data set was performed with *ML*2*D* and *CL*2*D* from the *Xmipp* package (Scheres *et al.*, 2005 ▶). Several starting models were generated from top-view and side-view averages applying *D*5 symmetry, as suggested by mass spectrometry, and were refined using the projection-matching algorithm in *Xmipp*. For the final reconstruction, only the 3117 particles corresponding to side views with parallel rings (see §[Sec sec3]3) were employed. The hand of the three-dimensional reconstruction was decided by comparison with available crystal structures of homologous proteins. The final resolution was estimated to be 20 Å using Fourier shell correlation (FSC; 0.5 correlation-coefficient cutoff). Fitting of atomic structures into the cryo-EM density was carried out using *UCSF Chimera* (Pettersen *et al.*, 2004 ▶).

### 2.4. Native mass spectrometry   

A 20 µl aliquot of the MtGSII-2a complex was buffer-exchanged into ammonium acetate buffer pH 7.5 using Micro Bio-Spin 6 columns (Bio-Rad). To acquire MS spectra under nondenaturing conditions, 2 µl aliquots were electrosprayed from gold-coated borosilicate capillaries prepared in-house. Spectra were recorded on a Q-Tof 2-type mass spectrometer (Waters) modified for high mass detection and adjusted to preserve noncovalent interactions (Hernández & Robinson, 2007 ▶). MS experiments were performed at a capillary voltage of 1700 V and a cone potential of 50 V. In experiments where high activation was employed, the collision energy was increased from 100 to 200 V. LC-MS analysis of the MtGSII-2a monomer was carried out on an NCS-3500RS nano-LC system (Thermo) equipped with a nano-UV detector set at 214 and 280 nm. The MtGSII-2a sample was prepared in solvent *A* (0.05% trifluoroacetic acid; TFA) and 1 µl was applied onto a nano PS-DVB reverse-phase monolithic column (100 µm internal diameter × 25 cm; Thermo) equilibrated with 90% solvent *A* and 10% solvent *B* (0.04% TFA, 90% acetonitrile). MtGSII-2a monomer was eluted with a linear gradient of 10–70% solvent *B* in 15 min at a flow rate of 600 nl min^−1^. The column effluent was delivered to a nanoflow ESI source and analyzed by MS on a QSTAR XL mass spectrometer (Applied Biosystems).

### 2.5. Analysis of enzyme-activity inhibition   

The activities of the His-tagged GS isoenzymes (GSII-1a, GSII-1b, GSII-2a and GSII-2b) were determined by quantifying the γ-glutamyl hydroxamate produced by the synthetase reaction (GSs) as described previously (Cullimore & Sims, 1981 ▶; Cullimore *et al.*, 1982 ▶). The reactions started by the addition of 10 µg purified His-tagged GS isoenzyme to the assay mixture (100 m*M* Tris buffer pH 7.8, 100 m*M* glutamate, 8 m*M* ATP, 8 m*M* hydroxylamine, 16 m*M* MgSO_4_) were allowed to proceed at 30°C for 15 min and were stopped by adding one volume of stop solution (0.37 *M* FeCl_3_, 0.67 *M* HCl, 0.2 *M* TCA). GS specific activity was determined as the amount of γ-glutamyl hydroxamate produced per minute per milligram of purified protein (µmol min^−1^ mg^−1^). GS inhibition was evaluated by adding increasing concentrations of either phosphinothricin (PPT; Fluka; 0, 0.005, 0.01, 0.05, 0.1, 0.5, 1, 1.5, 2.5, 5, 10 and 20 m*M*) or methionine-*S*-sulfoximine (MSO; Sigma; 0, 0.5, 1, 1.5, 2.5, 5, 10, 20, 40 and 60 m*M*) to the reaction mixture. GS inhibition assays were performed at two different glutamate concentrations (20 and 100 m*M*). IC_50_ was calculated by nonlinear regression using the LogIC50 equation of *GraphPad Prism*5 (GraphPad Software).

## 3. Results   

### 3.1. Differential inhibition of *M. truncatula* GSII isoenzymes   

**Figure 1:**
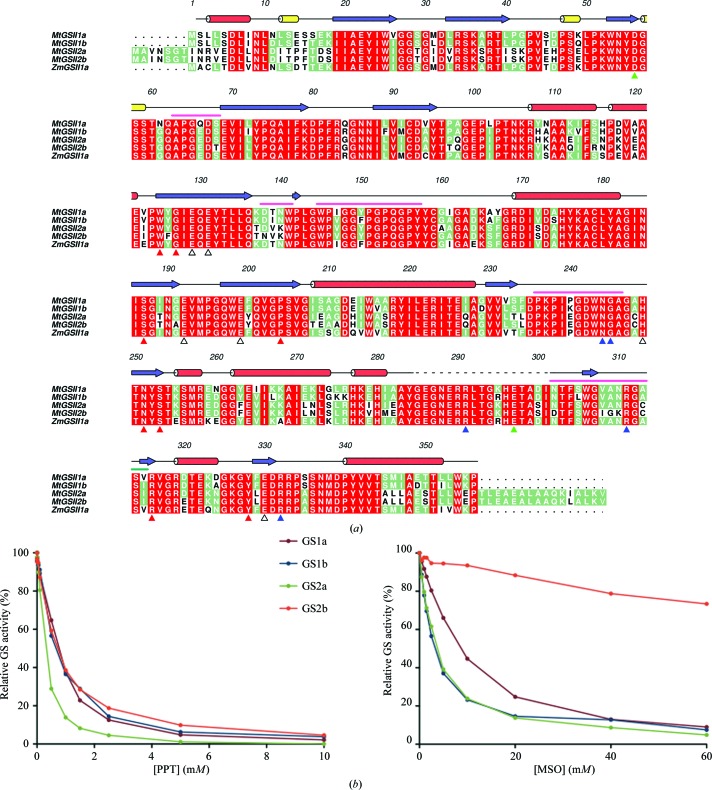
(*a*) Amino-acid sequence alignment of plant glutamine synthetases, including *M. truncatula* GSII-1a (MtGSII1a; UniProt O04998), GSII-1b (MtGSII1b; UniProt O04999), GSII-2a (MtGSII2a; UniProt Q84UC1), GSII-2b (MtGSII2b; UniProt E1ANG4) and *Z. mays* GSII-1a (ZmGSII1a; PDB entry 2d3a; Unno *et al.*, 2006 ▶; differing from UniProt B9TSW5 by a I353V replacement), performed with *ClustalW* (Larkin *et al.*, 2007 ▶). The transit peptides of *M. truncatula* GSII-2a and GSII-2b were omitted. Red, green and white backgrounds are used for high, medium and low conservation, respectively. Numbers above the alignment and secondary-structure elements (α-helices in red, 3_10_-helices in yellow and β-strands in blue) correspond to the crystal structure of MtGSII-1a described here. A dashed line denotes disordered residues in the crystallographic model. The ligand-contacting residues in *Z. mays* GSII-1a are indicated below the alignment with red (ATP), blue (glutamate), green (ammonia) or open (metal coordination) triangles. A pink line above the alignment indicates the structurally distinct regions between MtGSII-1a and ZmGSII-1a. Prepared with *Aline* (Bond & Schüttelkopf, 2009 ▶). (*b*) Inhibition of *M. truncatula* GSII by increasing concentrations of PPT (left) and MSO (right). The GS activity was normalized to that found in the absence of inhibitor. PPT and MSO were titrated using standard synthetase reactions with 100 or 20 m*M* glutamate, respectively.

The four *M. truncatula* isoenzymes are highly conserved, displaying 70–94% sequence identity (Fig. 1 ▶ *a*). Two small extensions (seven and 16 residues long at the N-terminus and the C-­terminus, respectively) distinguish the mature forms of *M. truncatula* plastid-located GS from the cytosolic counterparts. The dicotyledonous enzymes are also strikingly similar to the only structurally characterized monocotyledonous homologue, *Z. mays* GSII-1a (Unno *et al.*, 2006 ▶), with which they share 62–88% identical residues, including strict conservation of all of the amino acids that directly interact with the substrates (Fig. 1 ▶ *a*).

The susceptibility of each of the four *M. truncatula* GSII isoenzymes to inhibition by phosphinothricin (PPT) and methionine-*S*-sulfoximine (MSO) was evaluated (Fig. 1 ▶ *b*). The two compounds are glutamate analogues and have been shown to compete with this amino acid for binding to the active site (Ronzio *et al.*, 1969 ▶; Colanduoni & Villafranca, 1986 ▶; Manderscheid & Wild, 1986 ▶). PPT and MSO are phosphorylated in the presence of ATP, leading to the formation of a compound similar to the tetrahedral glutamate-adduct intermediate, resulting in irreversible inhibition of the enzyme (Liaw & Eisenberg, 1994 ▶). The percentage of GS inhibition caused by different concentrations of PPT or MSO was determined under standard GS assay conditions. The concentration of inhibitor reducing the GS activity by 50% (IC_50_) was estimated for PPT as 0.76, 0.67, 0.26 and 0.72 m*M* for GSII-1a, GSII-1b, GSII-2a and GSII-2b, respectively. For MSO titration (using 20 m*M* glutamate, given that the four GS isoenzymes were poorly inhibited under the standard GS assay conditions), the determined IC_50_ was 9.5, 2.2, 3.6 and 428.6 m*M* for GSII-1a, GSII-1b, GSII-2a and GSII-2b, respectively. Clearly, the four *M. truncatula* isoenzymes are more susceptible to inhibition by PPT than by MSO. Interestingly, GSII-2b was found to be extremely resistant to MSO inhibition.

### 3.2. The atomic structure of MtGSII-1a reveals a homodecameric quaternary arrangement   

**Figure 2:**
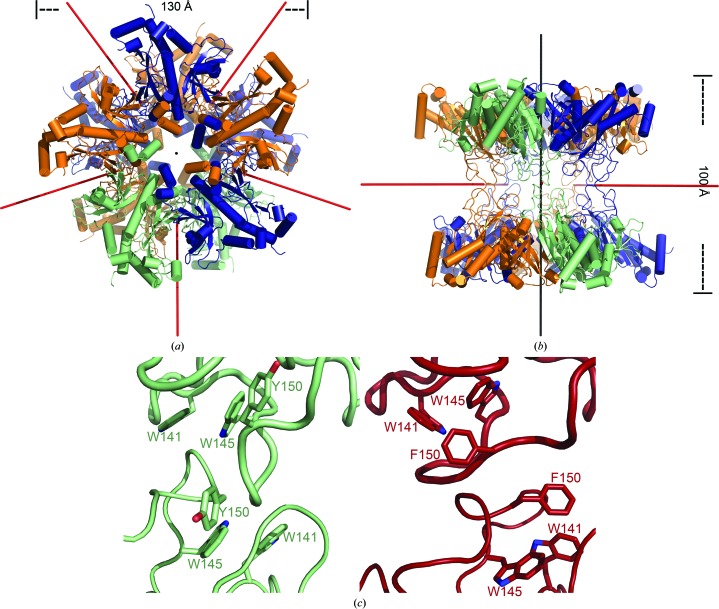
Three-dimensional structure of *M. truncatula* GSII-1a at atomic resolution. (*a*) Top view of the decameric assembly (*D*5 symmetry) along the fivefold symmetry axis (black line). *M. truncatula* GSII-1a subunits establishing inter-ring contacts are coloured in the same hue: green (monomers *A* and *F*), orange (monomers *B* and *G* and monomers *D* and *I*) or dark blue (monomers *C* and *H* and monomers *E* and *J*). The red lines indicate the positions of the five twofold axes. (*b*) Side view of the *M. truncatula* GSII-1a model resulting from a 90° rotation around *x* relative to the view in (*a*). Subunits are colour-coded as in (*a*), and the red and black lines represent the twofold and fivefold axes, respectively. (*c*) Close-up view of the inter-ring contact. In the left panel, *M. truncatula* GSII-1a subunits are coloured green (subunits *A* and *F*). The corresponding *Z. mays* GSII-1a subunits (subunits *A* and *I*) in the right panel are coloured red. The aromatic side chains involved in both interactions are labelled. In both molecules, a thicker ribbon represents subunit *A*.

The three-dimensional structure of *M. truncatula* GSII-1a was determined at 2.35 Å resolution by X-ray crystallography (Table 1 ▶) using synchrotron radiation (Seabra *et al.*, 2009 ▶). The crystallographic model revealed a decameric arrangement of the enzyme with *D*5 symmetry, with two stacked (face-to-face) pentameric rings. The pentameric rings of MtGSII-1a are ∼130 Å wide and stack to form a ∼100 Å tall prism-like structure (Figs. 2 ▶ *a* and 2 ▶ *b*). The ten MtGSII-1a monomers adopt very similar conformations, as highlighted by the r.m.s.d. of 0.35–0.65 Å for 291–311 aligned C^α^ atoms. As expected from the considerable sequence conservation (Fig. 1 ▶ *a*), *M. truncatula* GSII-1a is also structurally very similar to the *Z. mays* homologue (Unno *et al.*, 2006 ▶), with the superposed *A* subunits of both molecules displaying an r.m.s.d. of 1.00 Å for 321 aligned C^α^ atoms. In spite of this, however, six regions clearly deviate in these structures (Fig. 1 ▶ *a*, pink lines). Three of these structurally divergent sections are located at the inter-ring interface (Asp138–Trp141, Trp145–Tyr157 and Pro236–Ala246; *M. truncatula* numbering used throughout), while two others stretch into the active site (Ala63–Ser68 and Asn302-Val315). Also in the neighbourhood of the active site, the highly conserved segment encompassing Gly285-Ile301 is disordered in the crystallographic model of *M. truncatula* GSII-1a.

The extensive contacts established between the subunits that compose each ring result in a buried inter-monomer surface of approximately 3500 Å^2^. Additionally, each MtGSII-1a monomer establishes fairly limited interactions with just a single partner from the opposite ring. The overall buried inter-ring surface amounts to ∼2500 Å^2^, *i.e.* approximately 500 Å^2^ per individual monomer–monomer interaction, highlighting the weakness of the inter-ring contacts. Indeed, these contacts involve only the Trp141–Tyr150 segment of each of the intervening molecules (Fig. 2 ▶ *c*). Considering the contacting monomers *A* and *F* from opposed rings, hydrophobic interactions exist between the side chain of Trp141^*A*^ and both Ile147^*F*^ and Gly148^*F*^, as well as between Gly148^*A*^ and the side chain of Trp141^*F*^. Polar contacts are established between Trp145^*A*^ NE1 and Ile147^*F*^ O and between Gly148^*A*^ O and Trp145^*F*^ NE1. Finally, an additional hydrogen bond is formed between Tyr150^*A*^ N and Gly149^*F*^ O (Fig. 2 ▶ *c*). As a result of this particular arrangement of the inter-ring contacts, a 45° rotation around the fivefold axis of the molecules separates the chains of any given pair of contacting monomers (Figs. 2 ▶ *a* and 2 ▶ *b*).

The active sites of MtGSII-1a are located close to the intermonomer contacts within each oligomeric ring, similar to other glutamine synthetases. In this particular case, the structure of the apo form of the enzyme was determined and therefore there are no ligands present in any of the ten active centres. The segments Trp125–Glu131, Ser187–Glu192, Glu199–Pro204, Gly245–Ser253 and Tyr328–Arg332 delimit the funnel-shaped active-site cavity. The strictly conserved amino acids Glu129, Glu131, Glu192, Glu199, His249 and Glu330 (Fig. 1 ▶ *a*) are positioned approximately in the middle of this cavity, favourably located to mediate cation coordination. In all of the MtGSII-1a monomers a segment comprising the Thr293–Ala299 region, homologous to the Glu-binding loop of bacterial glutamine synthetases (Liaw & Eisenberg, 1994 ▶; Wray & Fisher, 2010 ▶), is disordered, leaving open the entrance to the glutamate-binding site at the narrower end of the active-site cavity.

### 3.3. MtGSII-2a is a homodecamer composed of two superposed pentameric rings   

**Figure 3:**
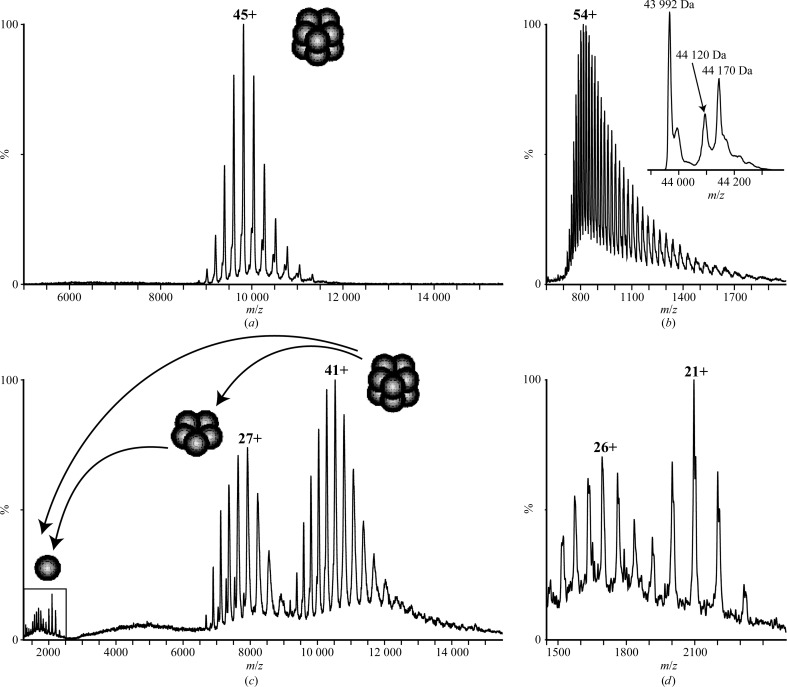
Nano-electrospray mass spectra of *M. truncatula* GSII-2a. (*a*) MS spectrum acquired for the intact MtGSII-2a assembly with a measured mass of 441 814 ± 75 Da, which is in good agreement with it existing as a decamer composed of ten copies of the *M. truncatula* GSII-2a monomer (*b*). (*c*) The spectrum recorded under harsh MS conditions is dominated by two charge-state series corresponding to the *M. truncatula* GSII-2a decamer (series centred at 41+) and pentamer (series centred at 27+), respectively, suggesting that the intact complex consists of two labile interacting pentamers. (*d*) Expansion of the *m*/*z* region below an *m*/*z* of 2500 in (*c*) showing *M. truncatula* GSII-2a monomers dissociated both from pentameric and decameric complexes in the gas phase.

The degree of sequence similarity of both MtGSII-2a and MtGSII-2b to other homodecameric GSIIs (Fig. 1 ▶ *a*) suggests that these plastid-located enzymes are also decameric. In order to confirm the oligomeric state of MtGSII-2a in solution, we recorded mass spectra of this protein under nondenaturing conditions. A single charge-state series at an *m*/*z* of around 10 000 (Fig. 3 ▶ *a*) dominates the spectrum with a measured mass of 441 814 ± 75 Da, in good agreement with the theoretical value of 441 235 Da for a decameric arrangement calculated from the monomer mass measured by LC-MS (Fig. 3 ▶ *b*). The slight increase in measured mass arises owing to incomplete desolvation of the assembly, a common phenomenon attributed to the retention of water and buffer molecules as a result of the ‘soft’ ionization methods required to maintain the integrity of the complex (McKay *et al.*, 2006 ▶). Interestingly, MS spectra acquired under high activation conditions revealed an additional charge-state series at a lower *m*/*z* region to the intact complex (Fig. 3 ▶ *c*). The mass measured for this series is 221 925 ± 10 Da, consistent with the presence of isolated pentamers under the stringent conditions under which mass spectra were recorded. Since all decameric GSII enzymes are suggested to organize as two pentameric rings (Unno *et al.*, 2006 ▶), this dissociation behaviour of MtGSII-2a in the gas phase has implications for the nature and strength of its subunit interactions, with stable bonding within each pentameric ring and a more labile and dynamic association between the double stacked rings (Benesch *et al.*, 2007 ▶; Hernández & Robinson, 2007 ▶). The less intense series below an *m*/*z* of 2500 (Figs. 3 ▶ *c* and 3 ▶ *d*) has a measured mass of 44 032 ± 51 Da, corresponding to monomeric MtGSII-2a dissociated from both MtGSII-2a pentamers and decamers in the gas phase. Overall, MS analysis of MtGSII-2a strongly supports a homodecameric organization of this enzyme in solution built as two stacked copies of a homopentameric ring.

### 3.4. Swinging motion of MtGSII-2a rings   

**Figure 4:**
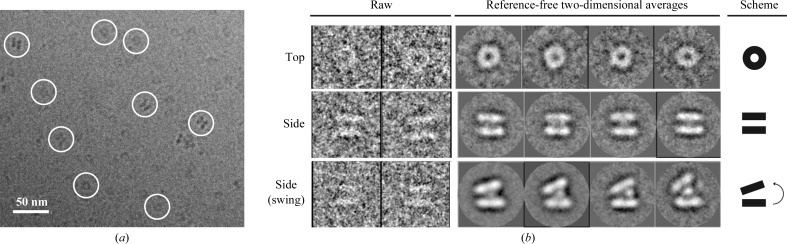
Electron cryomicroscopy images of *M. truncatula* GSII-2a. (*a*) EM field showing a homogeneous distribution of particles on the ice. (*b*) Comparison of the three major classes of particles observed in the data set, showing examples of single raw images (left) and reference-free two-dimensional class averages (middle). A schematic representation of the averages is shown for clarity (right).

Although the crystal structures of several GSII-1a enzymes have been obtained, all attempts to crystallize MtGSII-2a have so far been unsuccessful. To gain insight into the three-dimensional structure of this enzyme, we collected cryo-EM images under quasi-native conditions (Fig. 4 ▶ *a*). More than 7000 individual particles were selected and classified using reference-free two-dimensional alignment algorithms as implemented in *EMAN* (Ludtke *et al.*, 1999 ▶) and *Xmipp* (Scheres *et al.*, 2005 ▶). Two major classes were readily apparent in the data set, corresponding to the top and side views of the molecule, which are related by a 90° rotation (Fig. 4 ▶ *b*). In the top view, the pentameric ring expected from our native mass spectrometry results can be observed, although the symmetry is somewhat difficult to appreciate from two-dimensional averages (Fig. 4 ▶ *b*, top row). This is probably owing to the fact that the two rings are not perfectly aligned with respect to each other, as observed for all other GSII enzymes with known atomic structure. In the side view, two major and parallel masses of density can be identified which are connected by a central thinner mass corresponding to a weak inter-ring region (Fig. 4 ▶ *b*, central row). These views correlate well with the published structures of other GSII enzymes (Unno *et al.*, 2006 ▶; Krajewski *et al.*, 2008 ▶; He *et al.*, 2009 ▶) and also with the crystal structure of MtGSII-1a reported here (Figs. 2 ▶ *a* and 2 ▶ *b*).

Interestingly, we observed additional class averages in which the two masses of density that we attribute to the pentameric rings are no longer parallel (Fig. 4 ▶ *c*, bottom row). The sizes of the rings in these classes are identical to those observed in the standard side views, but the rings come as close as 20 Å on one edge, whereas they separate by up to 50 Å at the opposite end, generating a maximum tilt of 20°. In these averages, the central and weak mass observed in canonical side views is more prominent and is no longer located in the perfect centre of the ring mass, but rather condenses towards the edge where the two rings are more distant, allowing an approximation of the rings on the opposite edge. This swinging motion of the rings with respect to each other is likely to be owing to flexibility of the inter-ring connections. The presence of distinct subclasses in which the two rings are perfectly defined (Fig. 4 ▶ *b*, bottom row) suggests that non­parallel ring particles may reflect intermediate assembly stages in the formation/disruption of the enzyme decamer.

### 3.5. Electron cryomicroscopy structure of MtGSII-2   

**Figure 5:**
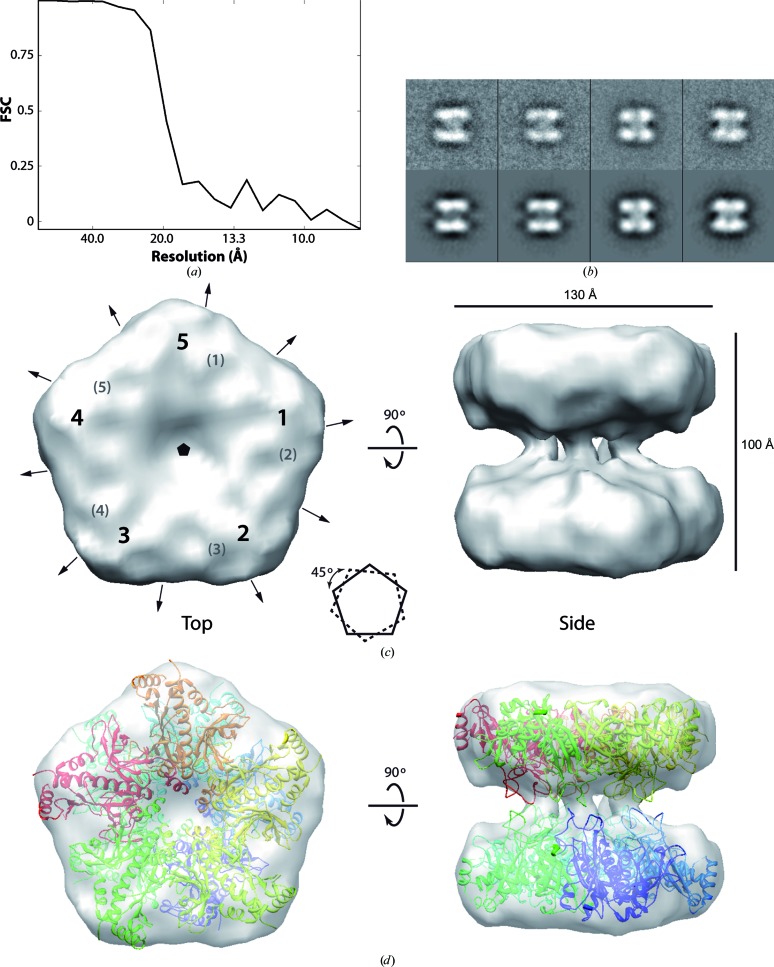
Electron cryomicroscopy reconstruction of *M. truncatula* GSII-2a. (*a*) FSC showing a resolution of 20 Å. (*b*) Pairs of class averages (upper row) and projections of the final three-dimensional reconstruction (bottom row). (*c*) Structure of *M. truncatula* GSII-2a, with approximate dimensions and positions of the symmetry axes (arrowed lines and pentagon for twofold and fivefold axes, respectively) defining the *D*5 symmetry. The numbers 1–5 and the numbers in parentheses indicate the positions of the five high-density blobs in the top and bottom rings, respectively. A scheme shows the relative rotation of the pentagons defined by the rings. (*d*) Fitting of the crystal structure of *M. truncatula* GSII-1a reported here, in which each monomer is coloured differently.

To deepen our understanding of the MtGSII-2a structure, we applied single-particle reconstruction methods to generate a three-dimensional model using the collected cryo-EM images. Because the mass spectra of MtGSII-2a strongly support a double pentameric organization, fivefold symmetry was applied during reconstruction procedures, using only particles in class averages corresponding to canonical side views (*i.e.* with parallel rings). Thus, the data set from which the reconstruction was calculated contains 3117 particles with fivefold symmetry. The final volume has a resolution of 20 Å (Fig. 5 ▶ *a*) based on Fourier shell correlation with a cutoff value of 0.5 and matches the experimental data well (Fig. 5 ▶ *b*). It represents the first structural information on a plastid-located GS enzyme.

The structure of MtGSII-2a has an overall cylindrical shape, with approximate dimensions of 100 Å in height and 130 Å in diameter (Fig. 5 ▶ *b*). It is formed by two parallel and symmetric rings, each composed of five monomers of the enzyme, as suggested by the five masses of globular density composing them (numbered 1–5 in Fig. 5 ▶ *c*). When observed from the top (*i.e.* along the fivefold symmetry axis), the pentagons defined by the two rings are rotated by ∼45° with respect to each other, which may have functional implications. Each of the rings is about 40 Å thick, leaving an inter-ring space of about 35 Å that is crossed at the centre by five threads of density connecting the rings. These threads are only 12 Å thick, which suggests that they could be flexible and thus rearrangements within them may provoke movement of the rings relative to each other. Fitting of the crystal structure of MtGSII-1a shows that the overall structure is very similar (Fig. 5 ▶ *d*), as expected from sequence alignment (Fig. 1 ▶ *a*). Similar results were obtained when the ZmGSII-1a crystal structure was fitted (not shown). Two distinct regions, comprising about 15% of the total enzyme mass, appear to protrude out of the density at the inter-ring space, likely owing to flexibility. The first region, comprising three loops (Ala63–Gly68, Pro236–Gly247 and Gly285–Asn302), is involved in interaction with the substrates, and thus these loops may only become ordered in their presence. The second region, comprising two loops (Gln136–Trp141 and Trp145–Gly155), forms the inter-ring connections that in our reconstruction seem to adopt a slightly different orientation to that observed in the crystal structure. Interestingly, both regions correspond to the areas that present different conformations in the two available crystal structures of plant GSII-1a. Although the resolution of our reconstruction does not allow atomic modelling of the active site, strict conservation of the residues involved in substrate recognition suggests that the structure of this region will be very similar to that observed for ZmGSII-1a, as discussed for GSII-1a.

## 4. Discussion   

In this report, we present the structures of two isoforms of *M. truncatula* GSII from different subcellular compartments determined using complementary experimental techniques. Cytosolic MtGSII-1a was characterized by X-ray crystallo­graphy and represents the first atomic structure of a dicotyledonous plant GSII enzyme, whereas the three-dimensional reconstruction of MtGSII-2a obtained by electron cryo­microscopy provides the first structural information on a plastid-located GSII. The two enzymes share a common decameric architecture, similar to the quaternary arrangement of other eukaryotic glutamine synthetases (Unno *et al.*, 2006 ▶; Krajewski *et al.*, 2008 ▶; He *et al.*, 2009 ▶), with two stacked antiparallel pentameric rings that interact through a limited surface region forming thin inter-ring connectors.

Despite the remarkable structural similarity between MtGSII-1a (r.m.s.d. of 0.98–1.03 Å for 320–322 aligned C^α^ atoms) and its closest structurally characterized homologue, ZmGSII-1a (Unno *et al.*, 2006 ▶), there are some critical differences between the two enzymes. Since MtGSII-1a was crystallized in the absence of substrates or inhibitors, a somewhat large region (Gly285–Ile301) that comprises the Glu-binding loop (Thr293–Ala299) is disordered. There is also a concerted movement of several segments of MtGSII-1a, notably the ∼180° rotation of Ala246, with its side chain now protruding into the glutamate-binding pocket, and the ∼90° rotation of the upstream Trp243 side chain that packs against Pro248 and leads to a rearrangement of the Lys237–Asp242 segment. Tyr150 now occupies the space left vacant by the Trp243 side chain, with concomitant reorganization of the inter-ring contact. Despite the near-perfect sequence conservation of the Trp141–Gly152 segment involved in establishing the interactions between the two pentameric rings of both enzymes (Fig. 1 ▶ *a*), the arrangement of this surface loop is dramatically different from ZmGSII-1a (Fig. 2 ▶ *c*). As a consequence, there is a difference of ∼15° in the relative rotation of the two superposed rings between the free MtGSII-1a and the inhibited ZmGSII-1a structures. It is therefore tempting to speculate that substrate binding and release by eukaryotic GS is connected to the rotation of the two homopentameric rings of the enzyme through the concerted movement of a limited set of regions in the vicinity of the active-site cleft and the inter-ring contact, and is in good agreement with the observed positive cooperativity of all isoforms of MtGSII (Seabra *et al.*, 2013 ▶).

The plasticity of the inter-ring contact is further underscored by a previously unidentified movement of the pentameric rings with respect to each other, as observed in cryo-EM images of MtGSII-2a. In this swinging motion, the rings almost contact on one side, breaking the perfect parallelism observed in all of the described crystal structures. We postulate that this phenomenon may be important for decamer assembly from two pentameric rings, as several different relative divergences of the rings are observed (Fig. 4 ▶ *b*, bottom row). This is possible because of rearrangements occurring at the level of the relatively weak inter-ring contacts, which are used as a hinge, as suggested above to explain the difference between the GSII-1a structures of *M. truncatula* and *Z. mays*. The fragility of the inter-ring contacts is supported by native mass-spectrometric data, showing that stringent mild conditions provoke the dissociation of decamers into pentamers. Recently, active pentameric GS rings have been detected by native gel electrophoresis in root nodules of *M. truncatula* (Seabra *et al.*, 2013 ▶), and GS dissociated active rings have also been reported to occur in the roots of *Beta vulgaris* (Mäck, 1998 ▶). According to the structural features reported here, there is no structural impairment of the preservation of catalytic activity in GS pentamers. However, differences in catalytic activity between the two oligomeric forms are predictable and could represent an additional mechanism of GS activity regulation in plant cells. The finding that active GS pentamers are exclusively detected in root nodules suggests differences in the assembly and/or stability of the enzyme in this particular organ, which is of particular interest.

The availability of the three-dimensional structures of closely related GS isoenzymes may be a valuable tool for the design of new herbicides. The four MtGSII isoenzymes differ in their catalytic properties (Seabra *et al.*, 2013 ▶) and, as shown here, in their susceptibility to inhibition by PPT or MSO. The IC_50_ values that we have determined for both inhibitors are in accordance with previous studies showing that, overall, plant GSII is 5–10 times more effectively inhibited by PPT than by MSO (Evstigneeva *et al.*, 2003 ▶). Nevertheless, while the four MtGSII isoenzymes showed a similar susceptibility to inhibition by PPT, their sensitivity to MSO is strikingly different (Fig. 1 ▶ *b*). The surprisingly elevated resistance of the MtGSII-2b iso­enzyme to MSO (IC_50_ of 428.6 m*M*) when compared with GSII-1a, GSII-1b and GSII-2a (IC_50_s of 9.5, 2.2 and 3.6 m*M*, respectively) is noteworthy.

**Figure 6:**
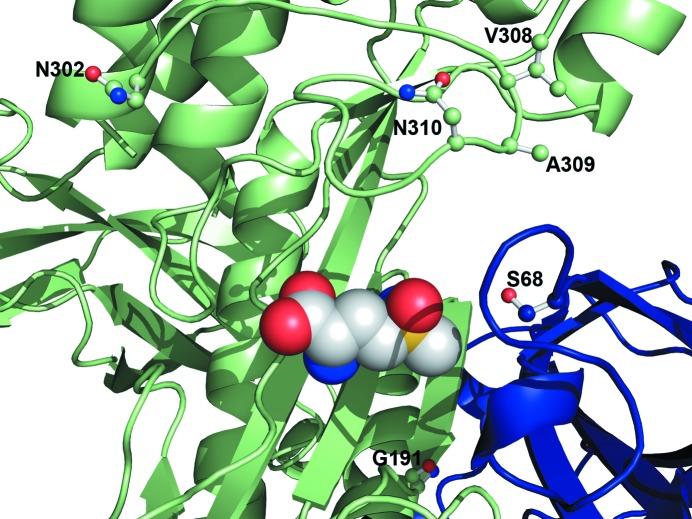
A small number of discrete amino-acid replacements give *M. truncatula* GSII-2b significant resistance to herbicide inhibition. The amino-acid sequence positions unique to *M. truncatula* GSII-2b and located in the vicinity of the MSO binding site are highlighted (stick representation) in the experimental model of *M. truncatula* GSII-1a. The active site at the interface between subunits *A* and *B* (colour code as in Fig. 2 ▶) is depicted and a methionine-*S*-sulfoximine molecule is shown in space-filling representation, as found in the complex with *Z. mays* GSII-1a (PDB entry 2d3b; Unno *et al.*, 2006 ▶).

This remarkable functional difference is likely to arise from unique amino-acid changes with respect to the other three isoforms, which in *M. truncatula* GSII-2b are limited to a few positions (Fig. 1 ▶ *a*). The majority of these substitutions are located on the enzyme surface and at a considerable distance from the active site, most likely being rather neutral. However, a few unique amino-acid changes occur in the vicinity of the MSO binding site and are therefore likely to modulate the sensitivity of *M. truncatula* GSII-2b to herbicides (Fig. 6 ▶). Notably, the Ile364-Gly365-Lys366 triplet (Val-Ala-Asn in all other *M. truncatula* GSII isoforms) precedes the invariable Arg367 (Arg311) involved in substrate binding, which in turn could establish contacts with the neighbouring Thr124 (Ser68). In close proximity, the strictly conserved Arg372 (Arg316), also a substrate ligand, leans against the Ile364-Gly365-Lys366 segment. Thus, its stabilization might be affected by the increased flexibility brought about by the replacement of an alanine by a glycine at position 365. Also, the metal-coordinating Glu248 (Glu192) is preceded by an alanine residue in *M. truncatula* GSII-2b, instead of the more flexible glycine found at this position in all other isoforms. Finally, and most strikingly, at one of the hinges of the glutamate-binding loop Asp358 (Asn302) is in close proximity to the invariable His333 (His277) and Lys334 (Lys278) side chains, and could therefore be involved in stabilizing the open conformation of the glutamate flap by establishing electrostatic interactions with these residues. The flexibility of the glutamate flap is essential for the catalytic activity of the enzyme, allowing the exchange of newly synthesized glutamine for glutamate. In its closed conformation, the glutamate-binding loop shields the reaction intermediates from solvent and harbours the essential Glu353 (Glu297) that binds the transition-state analogue arising from MSO phosphorylation (Liaw & Eisenberg, 1994 ▶). Therefore, stabilization of the open conformation of the glutamate-binding loop is expected to result in increased resistance to inhibition by MSO, as was observed in mutational studies of *Bacillus subtilis* GS, where changes in the glutamate flap led to up to 200-fold increased levels of resistance to inhibition by MSO (Wray & Fisher, 2010 ▶).

In conclusion, we have unveiled the quaternary architecture of dicotyledonous glutamine synthetase, and report a common decameric arrangement of cytoplasmic and plastid-located enzymes from the model legume *M. truncatula*. Both enzymes are composed of two juxtaposed homopentameric rings linked by a surprisingly flexible inter-ring contact. Moreover, we propose a zipper-like mechanism for ring–ring assembly, dependent on rearrangements of the inter-ring loops. Despite the structural similarity of the reported enzymes, there are striking differences in herbicide resistance among the distinct *M. truncatula* GS isoforms. The structural models now reported allowed the identification of a limited set of discrete and unique amino-acid substitutions in MtGSII-2b that account for its distinctive MSO resistance and pave the way for the design of herbicide-resistant plants.

## Supplementary Material

PDB reference: glutamine synthetase, 4is4

## Figures and Tables

**Table 1:** Data-collection and refinement statistics Values in parentheses are for the outermost resolution shell.

Data collection	
Space group	*P*2_1_
Unit-cell parameters (Å, °)	*a* = 99.3, *b* = 101.7, *c* = 188.1, β = 103.7
Resolution range (Å)	101–2.35 (2.48–2.35)
Reflections (measured/unique)	935718/149655
Completeness (%)	99.0 (96.8)
Multiplicity	6.3 (5.6)
*R*_merge_ †	0.077 (0.338)
Mean 〈*I*/σ(*I*)〉	19.2 (4.0)
Monomers per asymmetric unit	10
Matthews coefficient (Å^3^ Da^−1^)	2.2
Solvent content (%)	44.7
Refinement	
Resolution range (Å)	96.5–2.35
*R* factor‡/free *R* factor§ (%)	17.5/21.7
Unique reflections (working/test set)	141942/7518
Water molecules	969
Total No. of atoms	24909
R.m.s.d., bond lengths (Å)	0.008
R.m.s.d., bond angles (°)	1.165
Ramachandran plot statistics	
Residues in allowed regions (%)	100
DPI¶ (Å)	0.21

† *R*_merge_ = 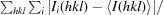 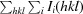, where *I*_*i*_(*hkl*) is the observed intensity and 〈*I*(*hkl*)〉 is the average intensity of multiple observations of symmetry-related reflections.

‡ *R* factor = 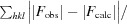 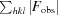, where |*F*_obs_| and |*F*_calc_| are the observed and calculated structure-factor amplitudes, respectively.

§ The free *R* factor is the cross-validation *R* factor computed for a randomly chosen subset of 5% of the total number of reflections which were not used during refinement.

¶ Diffraction-data precision indicator.

## References

[bb1] Afonine, P. V., Grosse-Kunstleve, R. W., Echols, N., Headd, J. J., Moriarty, N. W., Mustyakimov, M., Terwilliger, T. C., Urzhumtsev, A., Zwart, P. H. & Adams, P. D. (2012). *Acta Cryst.* D**68**, 352–367. 10.1107/S0907444912001308 PMC3322595 22505256

[bb2] Almassy, R. J., Janson, C. A., Hamlin, R., Xuong, N. H. & Eisenberg, D. (1986). *Nature (London)*, **323**, 304–309. 10.1038/323304a0 2876389

[bb3] Benesch, J. L., Ruotolo, B. T., Simmons, D. A. & Robinson, C. V. (2007). *Chem. Rev.* **107**, 3544–3567. 10.1021/cr068289b 17649985

[bb4] Bernard, S. M. & Habash, D. Z. (2009). *New Phytol.* **182**, 608–620. 10.1111/j.1469-8137.2009.02823.x 19422547

[bb5] Betti, M., García-Calderón, M., Pérez-Delgado, C. M., Credali, A., Estivill, G., Galván, F., Vega, J. M. & Márquez, A. J. (2012). *Int. J. Mol. Sci.* **13**, 7994–8024. 10.3390/ijms13077994 PMC3430217 22942686

[bb6] Bond, C. S. & Schüttelkopf, A. W. (2009). *Acta Cryst.* D**65**, 510–512. 10.1107/S0907444909007835 19390156

[bb7] Brunger, A. T. (2007). *Nature Protoc.* **2**, 2728–2733. 10.1038/nprot.2007.406 18007608

[bb8] Carlson, T. A. & Chelm, B. K. (1986). *Nature (London)*, **322**, 568–570.

[bb9] Carvalho, H. & Cullimore, J. (2003). *Recent Research Developments in Plant Molecular Biology*, edited by S. G. Pandalai, pp. 157–175. Trivandrum: Research Signpost.

[bb10] Carvalho, H., Lescure, N., de Billy, F., Chabaud, M., Lima, L., Salema, R. & Cullimore, J. (2000). *Plant Mol. Biol.* **42**, 741–756. 10.1023/a:1006304003770 10809446

[bb11] Carvalho, H., Lima, L., Lescure, N., Camut, S., Salema, R. & Cullimore, J. (2000). *Plant Sci.* **159**, 301–312. 10.1016/s0168-9452(00)00360-5 11074283

[bb12] Carvalho, H., Sunkel, C., Salema, R. & Cullimore, J. V. (1997). *Plant Mol. Biol.* **35**, 623–632. 10.1023/a:1005884304303 9349283

[bb13] Colanduoni, J. A. & Villafranca, J. J. (1986). *Bioorg. Chem.* **14**, 163–169.

[bb14] Cullimore, J. V., Lea, P. J. & Miflin, B. J. (1982). *Isr. J. Bot.* **31**, 155–162.

[bb15] Cullimore, J. V. & Sims, A. P. (1981). *Planta*, **150**, 392–396. 10.1007/BF00390175 24306889

[bb16] Doskočilová, A., Plíhal, O., Volc, J., Chumová, J., Kourová, H., Halada, P., Petrovská, B. & Binarová, P. (2011). *Planta*, **234**, 459–476. 10.1007/s00425-011-1419-7 21533644

[bb17] Dubochet, J., Adrian, M., Chang, J.-J., Homo, J.-C., Lepault, J., McDowall, A. W. & Schultz, P. (1988). *Q. Rev. Biophys.* **21**, 129–228. 10.1017/s0033583500004297 3043536

[bb18] Eisenberg, D., Gill, H. S., Pfluegl, G. M. & Rotstein, S. H. (2000). *Biochim. Biophys. Acta*, **1477**, 122–145. 10.1016/s0167-4838(99)00270-8 10708854

[bb19] Emsley, P., Lohkamp, B., Scott, W. G. & Cowtan, K. (2010). *Acta Cryst.* D**66**, 486–501. 10.1107/S0907444910007493 PMC2852313 20383002

[bb20] Evans, P. (2006). *Acta Cryst.* D**62**, 72–82. 10.1107/S0907444905036693 16369096

[bb21] Evstigneeva, Z. G., Solov’eva, N. A. & Sidel’nikova, L. I. (2003). *Appl. Biochem. Microbiol.* **39**, 539–543.

[bb22] Ghoshroy, S., Binder, M., Tartar, A. & Robertson, D. L. (2010). *BMC Evol. Biol.* **10**, 198. 10.1186/1471-2148-10-198 PMC2978018 20579371

[bb23] Gill, H. S., Pfluegl, G. M. & Eisenberg, D. (2002). *Biochemistry*, **41**, 9863–9872. 10.1021/bi020254s 12146952

[bb24] Goodman, H. J. & Woods, D. R. (1993). *J. Gen. Microbiol.* **139**, 1487–1493. 10.1099/00221287-139-7-1487 8103789

[bb25] He, Y.-X., Gui, L., Liu, Y.-Z., Du, Y., Zhou, Y., Li, P. & Zhou, C.-Z. (2009). *Proteins*, **76**, 249–254. 10.1002/prot.22403 19322816

[bb26] Hernández, H. & Robinson, C. V. (2007). *Nature Protoc.* **2**, 715–726. 10.1038/nprot.2007.73 17406634

[bb27] Krajewski, W. W., Collins, R., Holmberg-Schiavone, L., Jones, T. A., Karlberg, T. & Mowbray, S. L. (2008). *J. Mol. Biol.* **375**, 217–228. 10.1016/j.jmb.2007.10.029 18005987

[bb28] Kumada, Y., Benson, D. R., Hillemann, D., Hosted, T. J., Rochefort, D. A., Thompson, C. J., Wohlleben, W. & Tateno, Y. (1993). *Proc. Natl Acad. Sci. USA*, **90**, 3009–3013. 10.1073/pnas.90.7.3009 PMC46226 8096645

[bb29] Larkin, M. A., Blackshields, G., Brown, N. P., Chenna, R., McGettigan, P. A., McWilliam, H., Valentin, F., Wallace, I. M., Wilm, A., Lopez, R., Thompson, J. D., Gibson, T. J. & Higgins, D. G. (2007). *Bioinformatics*, **23**, 2947–2948. 10.1093/bioinformatics/btm404 17846036

[bb30] Lea, P. J. & Miflin, B. J. (2010). *Annu. Plant Rev.* **42**, 1–40.

[bb31] Leslie, A. G. W. & Powell, H. R. (2007). *Evolving Methods for Macromolecular Crystallography*, edited by R. J. Read & J. L. Sussman, pp. 41–51. Dordrecht: Springer.

[bb32] Liaw, S. H. & Eisenberg, D. (1994). *Biochemistry*, **33**, 675–681. 10.1021/bi00169a007 7904828

[bb33] Llorca, O., Betti, M., González, J. M., Valencia, A., Márquez, A. J. & Valpuesta, J. M. (2006). *J. Struct. Biol.* **156**, 469–479. 10.1016/j.jsb.2006.06.003 16884924

[bb34] Ludtke, S. J., Baldwin, P. R. & Chiu, W. (1999). *J. Struct. Biol.* **128**, 82–97. 10.1006/jsbi.1999.4174 10600563

[bb35] Mäck, G. (1998). *Planta*, **205**, 113–120. 10.1007/s004250050302 9599808

[bb36] Manderscheid, R. & Wild, A. (1986). *J. Plant Physiol.* **123**, 135–142.

[bb37] Mathis, R., Gamas, P., Meyer, Y. & Cullimore, J. V. (2000). *J. Mol. Evol.* **50**, 116–122. 10.1007/s002399910013 10684345

[bb38] McCoy, A. J., Grosse-Kunstleve, R. W., Adams, P. D., Winn, M. D., Storoni, L. C. & Read, R. J. (2007). *J. Appl. Cryst.* **40**, 658–674. 10.1107/S0021889807021206 PMC2483472 19461840

[bb39] McKay, A. R., Ruotolo, B. T., Ilag, L. L. & Robinson, C. V. (2006). *J. Am. Chem. Soc.* **128**, 11433–11442. 10.1021/ja061468q 16939266

[bb40] Melo, P. M., Lima, L. M., Santos, I. M., Carvalho, H. G. & Cullimore, J. V. (2003). *Plant Physiol.* **132**, 390–399. 10.1104/pp.102.016675 PMC166984 12746544

[bb41] Mindell, J. A. & Grigorieff, N. (2003). *J. Struct. Biol.* **142**, 334–347. 10.1016/s1047-8477(03)00069-8 12781660

[bb42] Pettersen, E. F., Goddard, T. D., Huang, C. C., Couch, G. S., Greenblatt, D. M., Meng, E. C. & Ferrin, T. E. (2004). *J. Comput. Chem.* **25**, 1605–1612. 10.1002/jcc.20084 15264254

[bb43] Reyes, J. C. & Florencio, F. J. (1994). *J. Bacteriol.* **176**, 1260–1267. 10.1128/jb.176.5.1260-1267.1994 PMC205187 7906687

[bb44] Ronzio, R. A., Rowe, W. B. & Meister, A. (1969). *Biochemistry*, **8**, 1066–1075. 10.1021/bi00831a038 4305484

[bb45] Rooyen, J. M. van, Abratt, V. R., Belrhali, H. & Sewell, T. (2011). *Structure*, **19**, 471–483. 10.1016/j.str.2011.02.001 21481771

[bb46] Scheres, S. H. W., Valle, M., Nuñez, R., Sorzano, C. O. S., Marabini, R., Herman, G. T. & Carazo, J. M. (2005). *J. Mol. Biol.* **348**, 139–149. 10.1016/j.jmb.2005.02.031 15808859

[bb47] Seabra, A. R., Carvalho, H. & Pereira, P. J. B. (2009). *Acta Cryst.* F**65**, 1309–1312. 10.1107/S1744309109047381 PMC2802889 20054137

[bb48] Seabra, A. R., Silva, L. S. & Carvalho, H. G. (2013). *BMC Plant Biol.* **13**, 137. 10.1186/1471-2229-13-137 PMC3848809 24053168

[bb49] Seabra, A. R., Vieira, C. P., Cullimore, J. V. & Carvalho, H. G. (2010). *BMC Plant Biol.* **10**, 183. 10.1186/1471-2229-10-183 PMC3095313 20723225

[bb50] Southern, J. A., Parker, J. R. & Woods, D. R. (1986). *J. Gen. Microbiol.* **132**, 2827–2835. 10.1099/00221287-132-10-2827 2887626

[bb51] Stanford, A. C., Larsen, K., Barker, D. G. & Cullimore, J. V. (1993). *Plant Physiol.* **103**, 73–81. 10.1104/pp.103.1.73 PMC158948 7516082

[bb52] Unno, H., Uchida, T., Sugawara, H., Kurisu, G., Sugiyama, T., Yamaya, T., Sakakibara, H., Hase, T. & Kusunoki, M. (2006). *J. Biol. Chem.* **281**, 29287–29296. 10.1074/jbc.M601497200 16829528

[bb53] Winn, M. D. *et al.* (2011). *Acta Cryst.* D**67**, 235–242.

[bb54] Woods, D. R. & Reid, S. J. (1993). *FEMS Microbiol. Rev.* **11**, 273–283. 10.1111/j.1574-6976.1993.tb00001.x 7691113

[bb55] Wray, L. V. Jr & Fisher, S. H. (2010). *J. Bacteriol.* **192**, 5018–5025. 10.1128/JB.00509-10 PMC2944536 20656908

[bb56] Wyatt, K., White, H. E., Wang, L., Bateman, O. A., Slingsby, C., Orlova, E. V. & Wistow, G. (2006). *Structure*, **14**, 1823–1834. 10.1016/j.str.2006.10.008 PMC1868402 17161372

